# 

**Published:** 1902-02

**Authors:** 


February, 1902. VOL. XXIII. No. 2.

### THE INTERNATIONAL DENTAL JOURNAL.

JAMES TRUMAN, D.D.S., EDITOR,
PHILADELPHIA.

*COLLABORATORS:*

CHARLES P. BRIGGS, A.B., M.D., D.M.D.,
BOSTON.

JOHN A. McCLAIN, D.D.S.,
PHILADELPHIA.

WILLIAM H. POTTER, A.B., D.M.D.,
BOSTON.

LIBRARY
SURGEON GENERAL'S OFFICE
6--FEB-1902

### Summary of Contents.

(*For details, see p. ii.*)

ORIGINAL COMMUNICATIONS.
REPORTS OF SOCIETY MEETINGS.
DOMESTIC CORRESPONDENCE.
EDITORIAL.
BIBLIOGRAPHY.
MISCELLANY.
CURRENT NEWS.

*$2.50 a Year, in Advance. Single Copies, 25 Cents.*

PUBLISHED MONTHLY BY

The International Dental Publication Company,

227-231 SOUTH SIXTH ST., PHILADELPHIA.

EDITORIAL OFFICE, 4505 CHESTER AVENUE, PHILADELPHIA, PA.

PRINTED BY J. B. LIPPINCOTT COMPANY, PHILADELPHIA.

"SCIENCE AS A TEACHER OF PROPHYLAXIS." Copyright, 1902, by SAMUEL A. HOPKINS, M.D., D.D.S., Boston, Mass.
Entered at the Post-Office at Philadelphia, and admitted for transmission through the mails at second-class rates.

